# Innovations in major system reconfiguration in England: a study of the effectiveness, acceptability and processes of implementation of two models of stroke care

**DOI:** 10.1186/1748-5908-8-5

**Published:** 2013-01-05

**Authors:** Naomi Fulop, Ruth Boaden, Rachael Hunter, Christopher McKevitt, Steve Morris, Nanik Pursani, Angus IG Ramsay, Anthony G Rudd, Pippa J Tyrrell, Charles DA Wolfe

**Affiliations:** 1 Department of Applied Health Research, University College London, 1-19 Torrington Place, London, WC1E 7HB, United Kingdom; 2 Manchester Business School, University of Manchester, Booth Street West, Manchester, M15 6PB, United Kingdom; 3 Research Department of Primary Care & Population Health, University College London, Royal Free Campus, London, NW3 2PF, United Kingdom; 4 Division of Health & Social Care Research, School of Medicine, King's College London, Capital House 7th Floor, 42 Weston Street, London, SE1 3QD, United Kingdom; 5 King’s College London Stroke Research Patients and Family Group, Division of Health & Social Care Research, School of Medicine, King's College London, Capital House 7th Floor, 42 Weston Street, London, SE1 3QD, United Kingdom; 6 Guy’s and St Thomas’ NHS Foundation Trust, St Thomas' Hospital, London, SE1 7EH, United Kingdom; 7 The University of Manchester Stroke & Vascular Centre, Manchester Academic Health Science Centre, Salford Royal Hospitals NHS Foundation Trust, Eccles Old Road, Stott Lane, Salford, M6 8HD, United Kingdom

**Keywords:** Stroke, Improvement science, Service reorganisation, Organisational change, Outcomes, Leadership, Implementation, Health services research, Cost effectiveness

## Abstract

**Background:**

Significant changes in provision of clinical care within the English National Health Service (NHS) have been discussed in recent years, with proposals to concentrate specialist services in fewer centres. Stroke is a major public health issue, accounting for over 10% of deaths in England and Wales, and much disability among survivors. Variations have been highlighted in stroke care, with many patients not receiving evidence-based care. To address these concerns, stroke services in London and Greater Manchester were reorganised, although different models were implemented. This study will analyse processes involved in making significant changes to stroke care services over a short time period, and the factors influencing these processes. We will examine whether the changes have delivered improvements in quality of care and patient outcomes; and, in light of this, whether the significant extra financial investment represented good value for money.

**Methods/design:**

This study brings together quantitative data on ‘what works and at what cost?’ with qualitative data on ‘understanding implementation and sustainability’ to understand major system change in two large conurbations in England. Data on processes of care and their outcomes (e.g. morbidity, mortality, and cost) will be analysed to evidence services’ performance before and after reconfiguration. The evaluation draws on theories related to the dissemination and sustainability of innovations and the ‘social matrix’ underlying processes of innovation. We will conduct a series of case studies based on stakeholder interviews and documentary analysis. These will identify drivers for change, how the reconfigurations were governed, developed, and implemented, and how they influenced service quality.

**Discussion:**

The research faces challenges due to: the different timings of the reconfigurations; the retrospective nature of the evaluation; and the current organisational turbulence in the English NHS. However, these issues reflect the realities of major systems change and its evaluation. The methods applied in the study have been selected to account for and learn from these complexities, and will provide useful lessons for future reconfigurations, both in stroke care and other specialties.

## Background

Significant changes in provision of clinical care within the English National Health Service (NHS) have been discussed in recent years, with the proposal to concentrate specialist services, such as major trauma, cardiac surgery, and specialist paediatrics, in fewer centres [1,2]. Such ‘reconfigurations’ have been defined as, ‘a deliberately induced change of some significance in the distribution of medical, surgical, diagnostic and ancillary specialties that are available in each hospital or other secondary or tertiary acute care unit in locality, region or healthcare administrative area’ [3].

Stroke is a major public health issue, accounting for over 10% of deaths in England and Wales, and much disability among stroke survivors [4,5]. Evidence indicated variations in quality of acute stroke care, as well as in services provided for the management of Transient Ischaemic Attack, rehabilitation, and life after stroke, with many patients not receiving evidence-based treatments [5,6]. The Department of Health’s National Stroke Strategy recommended major system change for stroke [4]. Recognising the need to improve stroke care at a system-wide level, London and Manchester led the way in this process. Other parts of the English NHS are now also seeking to reconfigure their stroke services [7].

Research has highlighted challenges in carrying out acute service reconfiguration [3,8,9], as has previous research on mergers of healthcare providers [10], particularly where there is resistance from professionals and the public [11]. Other research has highlighted challenges of major system change at local level [12]. This evaluation builds on previous research by studying the processes and outcomes of implementing system reconfigurations of acute stroke care in different contexts (London and Greater Manchester) and using different models. It also provides an opportunity to investigate health economic arguments about whether greater investment in acute stroke services might result in savings to the system overall [13].

### The reconfigurations

While there were numerous parallels between the London and Greater Manchester reconfigurations, there were also important differences.

Both reconfigurations were supported by local commissioners and received additional finance to support the changes. However, the London reconfiguration received significantly more resource per head of population: the London resource (£20 million) represented £2.42 per head of population (8.28 million [14]), and £1,816.13 per estimated annual stroke admission, whereas the Greater Manchester resource (£3.5 million) represented £1.56 per head of population (2.24 million [14]), and £1,174.81 per estimated annual stroke admission (Note estimates for stroke admission are derived from a peer-reviewed analysis of the South London Stroke Register [15]).

Both reconfigurations went through similar processes, including consultation, data analysis, model development, and specification and selection of services. However, approaches to leadership and governance of these processes differed: the changes in Manchester were led by the Greater Manchester and Cheshire Cardiac and Stroke Network (which facilitates stakeholder collaboration to improve commissioning and provision of stroke and cardiac care across the Greater Manchester area), whereas in London they were led by Healthcare for London, a programme within the city’s Strategic Health Authority (the organisation responsible for managing all NHS commissioning and provider organisations within London). Therefore, the London reconfiguration was led by a body with greater executive authority. The proposed changes in London and Manchester both met with a degree of resistance, for example, from local communities, service providers, and public representatives. The ways in which this was managed by reconfiguration leaders had a significant influence on how the models developed.

In terms of the models applied, prior to reconfiguration, in both London and Greater Manchester, patients presenting with stroke were taken to the nearest accident and emergency (A&E) department to receive stroke care. Both of the reconfigurations aimed to concentrate specialist stroke services in ‘hub and spoke’ models of provision. However, there were significant differences in these models, in terms of the services provided by the different levels of the new systems and the criteria applied in deciding where patients should be treated in the two systems.

**Figure 1:**
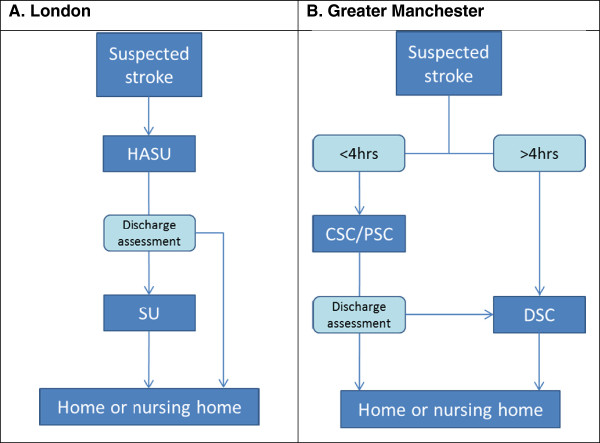
Simplified post-reconfiguration models in Greater Manchester and London.

In the reconfigured London model (Figure 1A), the local population is served by eight Hyperacute Stroke Units (HASUs), which provide immediate response to stroke, including assessment, stabilisation and any primary intervention; 24 Stroke Units, offering rehabilitation services; and 24 Transient Ischaemic Attack (TIA) services. Any person presenting with a suspected stroke is transferred to a HASU for assessment and treatment, then repatriated to a Stroke Unit, to a nursing home, or to their own home. In creating this system, a number of healthcare organisations lost either some or all of their stroke services (e.g. closure of stroke unit).

In the reconfigured Greater Manchester model (Figure 1B), the local population is served by 10 hospital organisations, or ‘trusts,’ providing District Stroke Centre (DSC) services; one Trust also hosts a Comprehensive Stroke Centre (CSC), which offers hyperacute stroke services in a neurosciences centre with access to interventional neuro-radiology and neurosurgery (24 hours per day, seven days per week); and two Trusts host Primary Stroke Centres (PSCs), providing thrombolysis (from 7 am to 7 pm, Monday to Friday). Any individual presenting within four hours of developing stroke symptoms is transferred to either the CSC or PSC for hyperacute care; once stable, he/she is repatriated either to a DSC, to a nursing home, or their own home. If presenting outside this four-hour ‘window,’ stroke patients in Greater Manchester are taken to the DSC to which they are nearest, much as they did prior to reconfiguration.

While there have been reconfigurations of acute services over recent years [16,17], there have been few similar examples within the NHS of services being completely restructured for such large populations over such a short period of time; and nowhere else in the world has attempted anything similar for stroke.

### Conceptual framework

These reconfigurations represent significant changes to the organisation and delivery of stroke services in London and Greater Manchester. A useful way to understand these reconfigurations is as processes of innovation. Reviews of the literature on the diffusion of innovations and major system change in healthcare draw attention to the need for more research on the processes by which such innovations are initiated (e.g. key drivers for change), implemented and sustained (or not), in what particular contexts [9,18]. This evaluation aims to contribute to the development of this evidence base by studying in depth the implementation of major service reconfiguration, using the example of stroke services.

We will study these innovations in two contrasting but complementary ways. First, we will take a more traditional health technology assessment approach to address ‘what works and at what cost?’ On its own, however, this approach pays little attention to structural pressures, e.g. professional pressures or processes whereby organisations innovate as a result of economic, regulatory, or legal reasons [19,20]. This approach also assumes innovation is always progressive and poor adopters are conservative, whereas resistance may be a rational response [21].

To understand how changes are implemented and sustained, the evaluation will draw on theories of adoption, diffusion, and sustainability of the innovation [18]; the characteristics of the innovation [22], and the role of local structural factors for example [23]. A review of evidence on diffusion of innovations [18] suggests sustainability relates to the nature of the innovation (e.g. the benefits it offers, how complex it is, how it is led, how stakeholders are involved, and use of evaluation and feedback) and the context into which it is introduced (local staff and organisational structures, inter-organisational networks, external pressures). We will also draw on Webster's concept of the ‘social matrix’ [21] originally applied to adoption of health technologies, where innovation is understood not as a technical, rational set of issues but in terms of requiring 'co-creation' by a range of stakeholders—where such processes create a 'social matrix' that is only as strong as the network of relations that hold it together. Studying the implementation of different models of system innovation in two contexts (London and Manchester) will contribute to understanding of the relationship between implementation and organisational context.

This evaluation was peer reviewed by the National Institute for Health Research prior to being funded (Project ref 10/1009/09), and received ethical approval in September 2011 from the London East NHS Research Ethics Committee (Ref 11/LO/1396).

### Study aims, objectives, and research questions

#### Aims

This study aims to use formative evaluation methods to support and analyse reconfiguration of acute stroke services in two regions of England and, in doing so, identify lessons that will guide future reconfiguration work in stroke and other services.

#### Objectives

1. To identify the barriers and facilitators for major system reconfiguration, implementation, and sustainability.

2. To study whether the reconfigurations have delivered clinical and cost effective improvements that patients and public think are worthwhile.

3. To identify lessons about major service reconfiguration that might be applied in other settings (i.e. other locations and other service domains).

#### Research questions

1. What were the key processes of and factors influencing the development and implementation of two reconfigurations of acute stroke services?

2. To what extent have changes delivered process and outcome improvements?

3. Have changes delivered improvements that stakeholders (e.g. commissioners, staff, patients and the public, and reconfiguration leads) think are worthwhile?

4. Have changes delivered value for money?

5. Has the additional investment in London provided better outcomes than those achieved for less resource in Manchester?

## Design/methods

### Design

#### Understanding what works and at what cost

Identifying what process and outcome changes have occurred, and at what cost, provides evidence about the extent to which the reconfigurations have succeeded in their objectives of changing the system of service provision and quality of care. This component of the evaluation will analyse documentary evidence to establish the models applied in London and Greater Manchester; it will also identify whether any changes in process and outcomes can be associated with these changes.

In assessing the nature and results of the two reconfigurations, the evaluation will apply a controlled before-and-after design [24]. This will compare the London and Manchester models in terms of the impact they had on processes, outcomes, and costs of care. In addition to comparing London with Greater Manchester pre- and post-reconfiguration, the analysis will make a wider comparison with the rest of England. This approach will permit observed changes to be analysed in the context of changes that take place in the rest of England over this period. A two-step approach will be taken to the analysis of outcomes: in the first instance, the impact of the reconfigurations on mortality and length of stay will be investigated; if evidence of impact on these is found, a more detailed cost-effectiveness analysis will be undertaken.

#### Understanding implementation and sustainability

To develop worthwhile lessons for future reconfigurations, it is important to establish not just whether process and outcome changes took place, but also how and why they occurred. This will be achieved through qualitative methods (documentary analysis and stakeholder interviews). These data will be used to explore themes drawn from the evaluation’s conceptual framework, and thus establish the relationships between activities in support of change, the context, the complex interactions between stakeholders, and perceived process and outcome changes.

#### Synthesis of approaches

A multi-method case study approach will be employed to draw together the learning from the approaches described above. The case study method permits development and testing of theories on how efforts to bring about change interact with the context in which they take place; a multiple case study approach allows the development and testing of theories in several contexts [25-27] (London and Greater Manchester), although more local contextual factors will also be considered: a series of local ‘service-level’ case studies will be conducted. These will reflect the main ‘trajectories’ experienced by organisations participating in changes of this kind, including developing new services, and closing existing services. Each case study will draw together evidence from the evaluation’s quantitative and qualitative components to develop and test theories on how change activities interacted with local contexts to produce the outcomes observed.

### Data collection

#### Understanding what works and at what cost

To establish what models were applied and how they were developed and put into action in London and Greater Manchester, the evaluation will analyse a range of documentary evidence. This will include project board papers, service designation criteria, and tariff documentation.

Using routinely collected data, the evaluation will assess whether the reconfigurations were associated with changes in process, outcomes, and costs of care. Data will be collected retrospectively from 2004, up to the point at which services began to change; while ‘after’ data will be collected from the points at which the new models were fully in place, through to December 2013.

Care process measures have been selected for the evidence indicating relevance to quality of care and their positive association with improved outcomes [28-30]. Measures include proportion of patients appropriately receiving thrombolysis, admitted to a stroke unit for 50% of their stay, appropriately receiving aspirin in the acute period, assessed by a multidisciplinary team, and receiving a swallowing test within the first 24 hours of care. These data will be drawn from national data sources (the Royal College of Physicians’ Sentinel Stroke Audit, the Stroke Improvement National Audit Programme (SINAP), and the Sentinel Stroke National Audit Programme (SSNAP)).

Outcome measures will include patient’s length of hospital stay, discharge destination, readmission rates, and mortality (in hospital, at 30 days, and at one year); these data will be drawn from HES and ONS datasets. Patient’s independent function (measured through Barthel Index score at discharge, and/or Modified Rankin score) will be drawn from Sentinel audit data, SINAP and SSNAP.

### Understanding implementation and sustainability

#### Documentary analysis

Documents covering the development and implementation of both reconfigurations will be collected [31,32]. These will include strategy documents (consultation, implementation), records of events held in support of the reconfigurations (e.g. public consultations); the proposals for change; and documentation of the governance of the reconfigurations (such as committees’ terms of reference and meeting minutes). Reflecting the evaluation’s interest in sustainability of change, such documentation will be collected over the lifespan of the study.

#### Interviews

This evaluation aims to use case study methods to provide an in-depth understanding of the factors influencing the process, implementation and impact of large scale reconfigurations, including how and why the changes took place. Methodological reviews indicate that interview and observation methods are significantly more effective than surveys in accessing such information [26,27].

The evaluations of the London and Greater Manchester reconfigurations will each be based on semi-structured interviews with the people who led, planned, and governed the changes, and draw together learning from the local ‘service-level’ case studies. We will conduct three such case studies in Greater Manchester and five in London.

In populating each ‘service-level’ case study, semi-structured interviews with the following informants will be conducted: representatives of senior and service-level management, doctors, and nurses in selected service provider organisations; senior representatives of commissioning organisations associated with the selected service provider organisations; and patients and carers who have recently received stroke care at the selected service provider organisations.

Interview data will be collected over two phases: Phase one will obtain views of the process and impact of reconfigurations, while phase two interviews will address longer term impact of the reconfigurations and incorporate findings from the first phase of interviews and the quantitative analyses.

#### Participants

Interviewees will be sampled purposively. Staff interviewees will be interviewed in both phases, while patient and carer interviews will be conducted with people who have recently experienced stroke services – i.e. the patients and carers interviewed in phase one and phase two will not be the same people.

#### Staff

For both sites, one-to-one interviews will be conducted with leaders of the reconfigurations, such as people who led or sat on the committees governing the reconfigurations (N = 20). Representatives of service provider organisations and associated commissioners will be interviewed to capture a range of experiences, including developing new hyperacute services, and closing established stroke services (N = 40). This should lead to a total of approximately 60 interviewees across the two sites in each phase.

#### Patients and carers

One-to-one interviews with patients or their carers will be conducted. We aim to interview patients unless they are unable to participate in an interview, in which case we would seek to interview the patient’s carer, instead. To ensure participants have recent experience of reconfigured stroke services, people will be approached and recruited just before discharge from hospital and will be interviewed approximately three months after discharge. Approximately eight patients or their carers will be interviewed per site, per phase, making a total of approximately 32 interviews. A sample of the patient and carer interviews will be conducted by a service user expert, who will receive formal social research methods training.

Over the two phases, then, a total of approximately 120 interviews with reconfiguration leads, commissioners and service providers, and approximately 32 interviews with patients and carers, will be conducted.

### Recruitment

#### Staff

Relevant staff interviewees will be identified in discussions with local stroke network leads and through documentary analysis, and will be limited to those who had some type of involvement in the reconfiguration process or the resultant changes to services. Potential staff participants will initially be approached by the study researchers. Contact, including provision of information sheets, will be made through e-mail and telephone.

#### Patients and carers

The approach to recruiting patients and carers is guided by the recruitment approach used by the South London Stroke Register [15]. Clinical staff will identify patients who are medically stable and close to discharge. A member of clinical staff will then ask these potential participants if they are willing to speak with a project researcher about the study. If the individual is willing, a study researcher will then verbally explain the purpose of the study, and provide written information. If the potential participant is still interested and agreeable, the researcher will allow at least 24 hours to elapse, then contact the potential participant again to ask for his/her agreement to participate in an interview. If the potential participant agrees, interviews will take place within three months of discharge, at a time and place mutually agreed with the patient and/or carer. The potential participant will be free to withdraw at any stage: when first approached, again when asked for agreement 24 hours later, and at any point subsequently, up to and during the actual interview.

#### Procedure

Interviews will take place in a private location agreed with the participant, or over the telephone.

Interviews with leaders of the reconfigurations will include the following topics: background to reconfigurations and catalysts for change, e.g. national and local factors; governance of the reconfigurations; development of the reconfigurations (establishing case for change, developing the models, populating the models); processes of implementation; impact on staff and services, including health and social services; impact on patients and the public; and contextual factors influencing the reconfiguration (e.g. finance and the organisational setting).

Interviews with local stroke services staff (including those in decommissioned services) and local commissioners will address the following topics: processes of implementation; impact on staff and services, including health and social services; impact on patients and the public; and contextual factors influencing the reconfiguration (e.g. finance and the organisational setting).

Interviews with patients and carers will address their experiences of the reconfigured stroke service, including: experiences of admission to hospital/stroke unit; staff communication around diagnosis; provision of information about condition, tests and treatments; how confident patients were that staff were knowledgeable; how patients’ problems were addressed; transfer through services; support in preparing to be discharged; and level of support since leaving services.

### Data analysis

#### Understanding what works and at what cost

Documentary evidence will inform the evaluation in terms of development and progress of the reconfigurations, e.g. providing detail on chronology, responsibilities and time dedicated to governing the change process [31,32].

The documentary analysis will establish the models applied in both reconfigurations. It will also provide information on key processes (e.g. governance activities, consultation events and designation of services). It will also contribute to understanding of the cost of change (by examining the work and time dedicated to governance, development, and implementation of the changes).

#### Analysis of process and outcome data

Routinely collected data will be analysed for two purposes. First, to analyse outcomes, econometric and cost effectiveness analyses will be conducted. Second, there will be an analysis of whether the reconfigurations are associated with significant changes in how services were provided, i.e. an analysis of process measures.

### Analysis of outcomes

#### Econometric analysis

The outcome variables measured at the Trust level in each time period will be regressed against the covariates, with particular interest in interactions showing the impact of region following the introduction of stroke service reconfigurations. The regression model is:

$$y_{\mathrm{it}}=\alpha_{0}+\alpha_{1}A_{i}+\alpha_{2}Y_{t}+\alpha_{3}R_{\mathrm{it}}+\alpha_{4}X_{\mathrm{it}}+e_{\mathrm{it}}$$

where *y* is the outcome of interest (mortality, LOS), *i* indicates Trust, *t* indicates year, *A* is region, *Y* is year, *R* is an indicator variable taking the value one if stroke services in Trust *i* in each region *A* in year *t* have been reconfigured (1 = yes, 0 otherwise), *X* is a set of patient and Trust characteristics, the αs are coefficients to be estimated, and *e* is an error term. The regression model used will depend on the nature of the dependent variable. Of particular interest are the sign and statistical significance of the coefficient α_*3*_. If for reconfigurations in London or Greater Manchester α_*3*_ indicates a favourable result, i.e. a reduction in mortality and/or on LOS, the following cost-effectiveness analysis will be undertaken.

#### Analysis of cost-effectiveness

A detailed Discrete Event Simulation model, with costs and outcomes summarised at 30 days, 90 days and 10 years, was developed to assess the cost effectiveness of the new London stroke model implemented in 2010 [33]. Following successful implementation of the model(s), and a sufficient period of time to collect adequate patient numbers, we will populate the model using the data sources mentioned above in *Data Collection*, for London and Greater Manchester ‘before’ and ‘after’ implementation. Costs will be assessed from the perspective of the NHS and personal social services (PSS). The proposed cost-effectiveness measure in the short run model is the incremental cost per death avoided at 90 days; in the long run it is the incremental cost per quality adjusted life year gained. Cost components will include: ambulance; scans; thrombolysis; length of stay on wards; and discharge destination. Cost-effectiveness will be calculated as the mean cost difference between the comparators divided by the mean difference in outcomes (90 day mortality/QALYs) to give the incremental cost-effectiveness ratio (ICER). We will undertake deterministic (one-, two- and multi-way) and probabilistic sensitivity analysis.

#### Analysis of functional independence

To analyse the effect of the reconfigurations on stroke patients’ functional independence, the regression model described for the econometric analysis of impact on outcomes will be applied to functional independence measures (e.g. Barthel Index score on discharge) where available, covering the same ‘before’ and ‘after’ periods.

#### Analysis of process changes

To analyse process changes, the regression model described for the econometric analysis of impact on outcomes will be applied to process variables, covering the same ‘before’ and ‘after’ periods. This will allow an assessment of whether relative performance across London and Greater Manchester remains constant pre- and post-reconfiguration – i.e. whether the reconfigurations are associated with changes in processes of care. Extending the ‘location’ comparison to include the rest of the English NHS will permit these changes to be assessed in the context of any nationwide changes that may have taken place over the period analysed.

#### Understanding implementation and sustainability

The case studies will be based on interview data, supported by documentary analysis. The documentary analysis will produce a detailed timeline of events in both reconfigurations. It will also identify potentially influential factors, including the types of justification used for change (e.g. research evidence, national policy, local service quality).

All interviews will be digitally recorded for transcription in full. Fieldwork notes will also be kept by the researcher. Data will be managed with NVIVO software. Iterative and thematic analysis of all data will be undertaken concurrently, according to well-established procedures of constant comparative analysis [34].

Interviews with governance, service provider, and commissioner informants will be analysed in terms of the theoretical framework suggested by innovations theory – i.e. the characteristics of the innovations, and the nature of the context into which they have been introduced—and in terms of Webster’s ‘social matrix’ theory: how the reconfigurations interacted with and influenced stakeholders’ networks and belief systems, for example regarding quality of care.

Interviews with patients or their carers will be analysed to address three objectives: to assess the novel components of the London and Manchester models (for example the transfer from HASU to SU in London); to assess whether issues prioritised by patients or their carers align with those raised in previous research on stroke care; and to support the formative component of the evaluation (e.g. through identifying local stroke patients’ care priorities).

Initial analysis and category building will be conducted by the researcher and will include category mapping and constant comparison. Validity will be assessed in relation to Patton’s four criteria of validity in qualitative research: verification, rival explanations, negative cases, and triangulation [35].

#### Data synthesis

As outlined above, this evaluation will bring together the learning from two complementary research strategies: ‘what works and at what cost’ and ‘understanding implementation and sustainability of change.’ The evaluation will seek to develop and test theories on how change activities interacted with contextual factors to result in the process and outcome changes observed.

In addition, the second phase of interviews will make use of initial qualitative and quantitative findings: data from the first phase of interviews, documentary analysis and the quantitative analysis, will be fed into interviews. This will permit an exploration of people’s views of reconfigurations, e.g. in light of evidence of impact on mortality and cost effectiveness.

The evaluation will draw together qualitative and quantitative data in this way for the overall London and Greater Manchester reconfigurations. Service-level case studies will be analysed to identify lessons relevant to particular reconfiguration ‘trajectories’ (e.g. creating new services, or closing existing ones) across varied settings.

## Discussion

The quantitative and qualitative components of the evaluation both provide learning relating to major system changes of this kind. The ‘what works and what cost’ component will identify quantifiable changes in provision of stroke services, and what impact these changes had. The ‘understanding implementation’ component allows identification of influential factors, such as key obstacles to and enablers of change, and how best to engage with these. The concept of the social matrix with its focus on the interactions and networks formed by stakeholders, and how these influence innovation – e.g. in identifying what is important, in developing and engaging in the innovation, and in influencing the definition of an innovation's ‘success’ (thus influencing potential sustainability) [21]—is likely to provide worthwhile lessons on the realities of negotiating change in complex settings.

In addition, combining the qualitative and quantitative research components, for example, by feeding quantitative findings into the second phase of interviews, will permit an examination of how change activities, contextual factors, and outcomes interrelate. The service-level case studies, which will focus on the various trajectories of change, will permit identification of both generalisable and context-specific lessons.

The evaluation faces a number of methodological and practical challenges: mainly, these derive from the timing of the reconfigurations—relative to one another, and to the evaluation itself; in addition, this evaluation takes place during a period of significant structural changes across the English NHS.

First, the reconfigurations took place at slightly different times, and took different trajectories, with Greater Manchester taking a more phased approach than occurred in London. Therefore, as the reconfigurations have different ‘before’ and ‘after’ phases, it is possible that external factors—such as participation in national audits—might have influenced the two reconfigurations differently. Also, conclusions drawn about the relationship between reconfiguration and any improvements seen are less clear in ‘before’ and ‘after’ studies. It is possible that any improvements that occur could have occurred anyway and not as a direct result of the policies implemented. Without any randomisation, it is also harder to rule out potential biases and gaming, such as admitting less complex patients. However, these complexities reflect the ‘real world’ nature of service reconfiguration, and change more broadly, and are thus likely to be faced quite commonly in evaluations of this kind. As our research design actively seeks to analyse such contextual influences, this in fact represents an opportunity to improve understanding of the relationship between national (‘macro’) drivers for improvement and organisational (‘meso’) responses to these.

The retrospective nature of this evaluation impacts on the qualitative component of the research. For example, planning and implementation events could not be observed; in addition, interviewees’ recollection of events will naturally vary. Ensuring suitable representation of stakeholders—based on documentary analysis—and applying Patton’s validity criteria [35] in the analysis will be key to ensuring that important lessons are not missed. In terms of studying patient experience, only patients who have recently experienced reconfigured services will be interviewed, so that no ‘before’ equivalent will be available. However, we will compare these patient experience data with literature on the experiences of stroke patients and carers [36,37], so that the analysis can examine whether patients’ concerns have been addressed by changes brought about by the reconfigurations.

This evaluation takes place amid the current turbulence prompted by the abolition of organisations directly involved in the reconfigurations, such as Strategic Health Authorities and Primary Care Trusts, and the planned reorganisation of Cardiac and Stroke Networks. This has the potential to limit our access to people working in these organisations. To ensure these perspectives are captured, the study timetable was altered after funding was awarded, so that interviews with these stakeholders could commence earlier than originally planned. Further, the methods will allow us to capture some of the influences of such turbulence, and how these might be dealt with when sustaining change. The distraction and uncertainty brought by changes in associated organisations is common in many settings, meaning the research may have relevance in a wide range of settings.

Finally, it is likely that reconfigurations of this kind will become increasingly common, not just in stroke care, but in other healthcare priorities, for example cardiac and vascular surgery, and major trauma [38]. Reviews indicate that more research is needed to understand drivers, processes, and outcomes when implementing such large-scale changes [9,18]. This evaluation’s theoretical framework offers the potential to contribute significant lessons on such matters, with relevance both within the UK, and internationally.

## Competing interests

NF, RB, RH, CM, SM, NP, AIGR, and CDAW have no competing interests. AGR is clinical lead for stroke in London, and provided clinical leadership for the London reconfiguration. PJT is clinical lead for stroke in Greater Manchester, and provided clinical leadership for the Greater Manchester reconfiguration.

## Authors' contribution

NF led overall study design and drafting of the protocol. RB, RH, CM, SM, NP, AIGR, AGR, PJT, and CDAW contributed to study design and drafting of the protocol. AGR led drafting of the London background section. PJT led drafting of the Greater Manchester background section. RH and SM led drafting of the quantitative design and analysis sections. NF, CM and AIGR led drafting of the qualitative design and analysis sections. All authors read and approved the final manuscript.
